# Improved image quality in abdominal computed tomography reconstructed with a novel Deep Learning Image Reconstruction technique – initial clinical experience

**DOI:** 10.1177/20584601211008391

**Published:** 2021-04-09

**Authors:** Tormund Njølstad, Anselm Schulz, Johannes C Godt, Helga M Brøgger, Cathrine K Johansen, Hilde K Andersen, Anne Catrine T Martinsen

**Affiliations:** 1 Department of Radiology and Nuclear Medicine, Oslo University Hospital Ullevål, Oslo, Norway; 2 Department of Diagnostic Physics, Oslo University Hospital, Oslo, Norway; 3 Department of Radiology, Haukeland University Hospital, Bergen, Norway; 4 Faculty of Health Sciences, Oslo Metropolitan University, Oslo, Norway

**Keywords:** Abdominal computed tomography, deep learning image reconstruction, image quality

## Abstract

**Background:**

A novel Deep Learning Image Reconstruction (DLIR) technique for computed tomography has recently received clinical approval.

**Purpose:**

To assess image quality in abdominal computed tomography reconstructed with DLIR, and compare with standardly applied iterative reconstruction.

**Material and methods:**

Ten abdominal computed tomography scans were reconstructed with iterative reconstruction and DLIR of medium and high strength, with 0.625 mm and 2.5 mm slice thickness. Image quality was assessed using eight visual grading criteria in a side-by-side comparative setting. All series were presented twice to evaluate intraobserver agreement. Reader scores were compared using univariate logistic regression. Image noise and contrast-to-noise ratio were calculated for quantitative analyses.

**Results:**

For 2.5 mm slice thickness, DLIR images were more frequently perceived as equal or better than iterative reconstruction across all visual grading criteria (for both DLIR of medium and high strength, p < 0.001). Correspondingly, DLIR images were more frequently perceived as better (as opposed to equal or in favor of iterative reconstruction) for visual reproduction of liver parenchyma, intrahepatic vascular structures as well as overall impression of image noise and texture (p < 0.001). This improved image quality was also observed for 0.625 mm slice images reconstructed with DLIR of high strength when directly comparing to traditional iterative reconstruction in 2.5 mm slices. Image noise was significantly lower and contrast-to-noise ratio measurements significantly higher for images reconstructed with DLIR compared to iterative reconstruction (p < 0.01).

**Conclusions:**

Abdominal computed tomography images reconstructed using a DLIR technique shows improved image quality when compared to standardly applied iterative reconstruction across a variety of clinical image quality criteria.

## Introduction

Computed tomography (CT) has become an indispensable clinical tool in contemporary medicine, with widespread availability, demonstrated safety, and non-invasive ability to rapidly image large anatomical volumes.^1^ This has fueled a rapid increase in the use of CT imaging over the last decades.^2^ Conjoint with the associated raise in radiation exposure, a concern towards the increased risk for radiation-induced malignancy has emerged.^3^ In general, the potential for dose-reduction is constrained by the radiologist ability to perform the given diagnostic task for which the CT examination was requested, where the benefit of dose-reduction is offset by an increase of image noise and deterioration of image quality.^4^ Thus, novel noise-reduction methods are aspired in clinical practice to improve image quality and diagnostic performance in pursuit of dose reduction.

Filtered back projection (FBP) has been the historical standard for CT image reconstruction, later accompanied by iterative reconstruction (IR) algorithms.^5–7^ However, recent concerns have been raised that dose-reduction in IR may deteriorate image quality due to a shift in image texture, primarily affecting low-contrast tasks.^8^ Recently, a novel engine based on a Deep Learning Image Reconstruction (DLIR) technique for CT was clinically approved in the US (TrueFidelity, GE Healthcare). Other vendor-specific algorithms for deep learning reconstruction are also emerging (AiCE, Canon Medical Systems). In brief, as explained by a technical white paper,^9^ the DLIR technique leverages a deep neural network-based engine to generate CT images from the X-ray projection sinogram. The algorithm has been trained with high-dose, high-quality FBP datasets on a large number of phantom and patient cases, covering variations in body composition, anatomies and clinical indications in order to learn how to suppress image noise without compromising image quality.

Noteworthy, although novel noise reduction methods (such as DLIR) may seem promising, it is imperative that they are comprehensively evaluated in phantom and clinical studies to ensure that established clinical benefits are not compromised.^8^ Preliminary investigations have shown that DLIR improves perceived *overall image quality* in abdominal CT.^10^ However, specific anatomical criteria have not been applied, such as the European guidelines on quality criteria for CT.^11^ Furthermore, there may be benefit of enhanced spatial resolution in images reconstructed in thinner slices than conventional thicker slices.^12^

On this basis, this study set out to investigate the image quality of abdominal CT images reconstructed with DLIR in both 0.625 mm and 2.5 mm slices compared to standardly applied IR across a selection of image quality metrics.

## Material and Methods

This study was conducted as part of a quality control initiative at our hospital, where 10 patients were retrospectively included having recently undergone a whole-body CT as part of routine follow-up for an underlying malignant condition. The study was approved by the institutional review board, and as all scans were clinically indicated and none merely performed for the purpose of this study, the need for individual patient consent was waived.

### Image acquisition and reconstruction

All CT scans were performed on a GE Revolution CT scanner (GE Healthcare), with standardly applied scanning parameters for whole-body CT (thorax, abdomen and pelvis) with contrast enhancement in the portal venous phase. Intravenous contrast with iohexol 350 mg/ml (Omnipaque, GE Healthcare) was administered based on patient weight (2 ml/kg), and with a fixed injection speed of 4 ml/s and scanner acquisition delay of 85 s. Scans were obtained using 120 peak kilovoltage (kVp), 8 cm detector collimation (128 × 0.625 mm), pitch of 0.5, rotation time 0.5 s, and large scan field of view (50 cm). Raw data were used to generate six axial image sets, applying standard IR (ASiR-V 50%, GE Healthcare), DLIR of medium strength, and DLIR of high strength (TrueFidelity, GE Healthcare) to generate images of both 0.625 mm and 2.5 mm slice thickness.

### Qualitative image quality analysis

Six different image sets for side-by-side comparison were created, as listed in Table 1. Images of each patient was presented twice to evaluate intraobserver agreement. Thus, a total of 120 hangings per reader were arranged in a randomized order, where images with DLIR and IR were randomly selected to be on the right and left monitor to avoid situation bias. Readers were blinded to study setup and reconstruction method applied, and all identifying patient information and annotations had been removed. Images were viewed under standard clinical conditions using a two-monitor high-resolution PACS workstation (Sectra IDS7, Sectra AB) where readers were free to scroll through images and adjust window level as needed to simulate a clinical setting. There was no time constraint for review.

**Table 1.** Overview of image sets for side-by-side visual comparison, evaluating image quality of Deep Learning Image Reconstruction (DLIR) of medium and high strength compared to Iterative Reconstruction (IR) for both 0.625 mm and 2.5 mm slice thickness.

	Monitor 1^a^		Monitor 2^a^		
No	Reconstruction technique	Slice thickness	Reconstruction technique	Slice thickness	No. of hangings per reader^b^
1	DLIR of medium strength	0.625 mm	IR	2.5 mm	20
2	DLIR of medium strength	0.625 mm	IR	0.625 mm	20
3	DLIR of medium strength	2.5 mm	IR	2.5 mm	20
4	DLIR of high strength	0.625 mm	IR	2.5 mm	20
5	DLIR of high strength	0.625 mm	IR	0.625 mm	20
6	DLIR of high strength	2.5 mm	IR	2.5 mm	20
Total					120

DLIR: Deep Learning Image Reconstruction; IR: Image Reconstruction.

^a^Images with DLIR and IR were randomly selected to be on the right and left monitor to avoid situation bias.

^b^Each of the 10 patients were presented twice to evaluate intraobserver agreement.

Image quality was assessed independently by three board-certified radiologists, where readers had a mean radiology experience of 16 years (range 12–24 years). Readers were presented with the image sets in a side-by-side comparative setting, assessing images along eight visual grading criteria as listed on the left-hand side in Tables 2 and 3. The first five criteria (C1–C5) were adapted and slightly modified anatomical criteria from the European guidelines for image quality in abdominal CT,^11^ and supplemented by assessment of image noise (C6), image texture (i.e. image coarseness) (C7), and image artifacts (C8), tailored to the purpose of this study. The images were compared on an ordinal five-point Likert-type scale, spanning from a score of –2 (images on the left monitor are clearly better) to +2 (images on the right monitor are clearly better), as shown in Table 4.

**Table 2.** Proportion of reader scores evaluating images reconstructed with Deep Learning Image Reconstruction (DLIR) as *equal*, *slightly better* or *clearly better* compared to images reconstructed with Iterative Reconstruction (IR).

		Reconstruction technique, slice thickness and DLIR strength compared					
		DLIR 0.625 mm versus IR 2.5 mm		DLIR 0.625 mm versus IR 0.625 mm		DLIR 2.5 mm versus IR 2.5 mm	
Visual grading criteria		Medium	High	Medium	High	Medium	High
C1	Visually sharp reproduction of the liver parenchyma	57%	98%***	100%^a^	100%^a^	100%^a^	97%***
C2	Visually sharp reproduction of the intrahepatic vascular structures	68%	98%***	100%^a^	100%^a^	98%***	97%***
C3	Visually sharp reproduction of the common bile duct in the pancreas	90%***	97%***	100%^a^	100%^a^	100%^a^	93%***
C4	Visually sharp reproduction of the origin of the superior mesenteric artery	67%*	97%***	100%^a^	100%^a^	97%***	95%***
C5	Visually sharp reproduction of the contours of the right adrenal gland	75%***	97%***	98%***	100%^a^	100%^a^	97%***
C6	Overall impression of image noise	53%	98%***	98%***	100%^a^	100%^a^	97%***
C7	Overall impression of image texture	72%**	97%***	98%***	98%***	98%***	97%***
C8	Overall impression of image artifacts	95%***	100%^a^	100%^a^	100%^a^	98%***	98%***

P-values by univariate logistic regression analysis. DLIR: Deep Learning Image Reconstruction; IR: Iterative Reconstruction.

^a^Not applicable – no p-value estimate as all observations were reported as *equal*, *slightly better* or *clearly better* in favor of DLIR.

*p < 0.05.

**p < 0.01.

***p < 0.001.

**Table 3.** Proportion of reader scores evaluating images reconstructed with Deep Learning Image Reconstruction (DLIR) as *slightly better* or *clearly better* as opposed to *equal* or in favor of images reconstructed with Iterative Reconstruction (IR).

		Reconstruction technique, slice thickness (mm) and DLIR strength compared					
		DLIR 0.625 mm versus IR 2.5 mm		DLIR 0.625 mm versus IR 0.625 mm		DLIR 2.5 mm versus IR 2.5 mm	
Visual grading criteria		Medium	High	Medium	High	Medium	High
C1	Visually sharp reproduction of the liver parenchyma	32%	85%***	92%***	100%^a^	98%***	97%***
C2	Visually sharp reproduction of the intrahepatic vascular structures	30%	63%*	77%***	97%***	77%***	97%***
C3	Visually sharp reproduction of the common bile duct in the pancreas	22%	35%	47%	73%**	53%	57%
C4	Visually sharp reproduction of the origin of the superior mesenteric artery	17%	43%	63%*	100%^a^	55%	75%***
C5	Visually sharp reproduction of the contours of the right adrenal gland	17%	42%	60%	92%***	57%	77%***
C6	Overall impression of image noise	33%	95%***	97%***	100%^a^	95%***	97%***
C7	Overall impression of image texture	32%	75%***	87%***	98%***	83%***	95%***
C8	Overall impression of image artifacts	10%	25%	32%	70%**	37%	50%

P-values by univariate logistic regression analysis. DLIR: Deep Learning Image Reconstruction; IR: Iterative Reconstruction.

^a^Not applicable – no p-value estimate as all observations were reported as *slightly* or *clearly better* for DLIR.

*p-value in favor of DLIR < 0.05.

**p-value in favor of DLIR < 0.01.

***p-value in favor of DLIR < 0.001.

**Table 4.** Overview of ordinal five-point scale used for visual grading in the image quality assessment.

Score	Description
–2	Images on the left monitor are clearly better
–1	Images on the left monitor are slightly better
0	The images on the left and right monitor are equally good
+1	Images on the right monitor are slightly better
+2	Images on the right monitor are clearly better

### Quantitative image quality analysis

For quantitative image analysis, a quadratic region of interest (ROI) measuring 11 by 11 pixels was placed in the portal vein, adjacent normal liver parenchyma and aorta for all 10 patients using a tailored script in the Matlab environment (Matlab version R2018a, MathWorks). For each ROI, care was taken to avoid confounding structures, such as vessels, lesions and plaques. The script ensured that ROI locations were identical across reconstructions for the same patient. For each reconstruction, mean and standard deviation in CT numbers (Hounsfield Units, HU) were recorded within each ROI, where the latter was regarded as a marker of image noise. Contrast-to-noise ratio (CNR) was computed by the following formula, in accordance with Thitaikumar et al.^13^

$$\mathrm{CNR}=\frac{2{\left(s_{1}-s_{2}\right)}^{2}}{\sigma_{1}^{2}+\sigma_{2}^{2}}$$

where *s* denotes the signal (mean CT number) and σ the standard deviation (noise), and subscripts 1 and 2 represent the two target ROIs (portal vein and liver parenchyma, respectively).

### Statistical analysis

Study data were recorded and processed using Microsoft Office Excel 2010 (Microsoft Corporation) and evaluated using IBM SPSS Statistics version 26.0 (IBM Corporation). Patient demographics, patient size and scanner dose were summarized as mean, minimum and maximum. For quantitative analyses, difference in CT-numbers, noise and CNR were evaluated by the Student’s *t*-test. For qualitative analyses, the distribution of Likert scale reader scores across reconstructions was displayed using bar charts. Categorized scores were evaluated using univariate logistic regression. Interobserver agreement was evaluated with the intraclass correlation coefficient, ICC(2,k), as defined by Shrout and Fleiss.^14^ ICC was estimated including all scores across the 10 patients and eight visual grading criteria (a total of 960 scores per reader, as each patient was evaluated twice). ICC intervals were interpreted according to Koo and Li, with ICC < 0.5 indicating poor reliability, 0.51–0.75 moderate reliability, 0.76–0.9 good reliability and 0.90–1.00 excellent reliability.^15^ Intraobserver agreement was calculated with κ statistics, where κ was interpreted according to Landis and Koch with κ < 0 indicating poor agreement, 0.00–0.20 slight agreement, 0.21–0.40 fair agreement, 0.41–0.60 moderate agreement, 0.61–0.80 substantial agreement and 0.81–1.00 almost perfect agreement.^16^

## Results

Among the 10 patients included, there were three men and seven women. Mean age was 67.7 years (range 40–91 years). Mean anteroposterior diameter was 25.6 cm (range 19.5–33.3 cm) and mean width was 32.2 cm (range 23.7–39.9 cm). Volume CT dose index (CTDIvol) was reported to a mean of 14.7 mGy (range 8.3–27.0 mGy) and dose-length product (DLP) to 1087 mGy⋅cm (range 602–2212 mGy⋅cm).

**Fig. 1.**
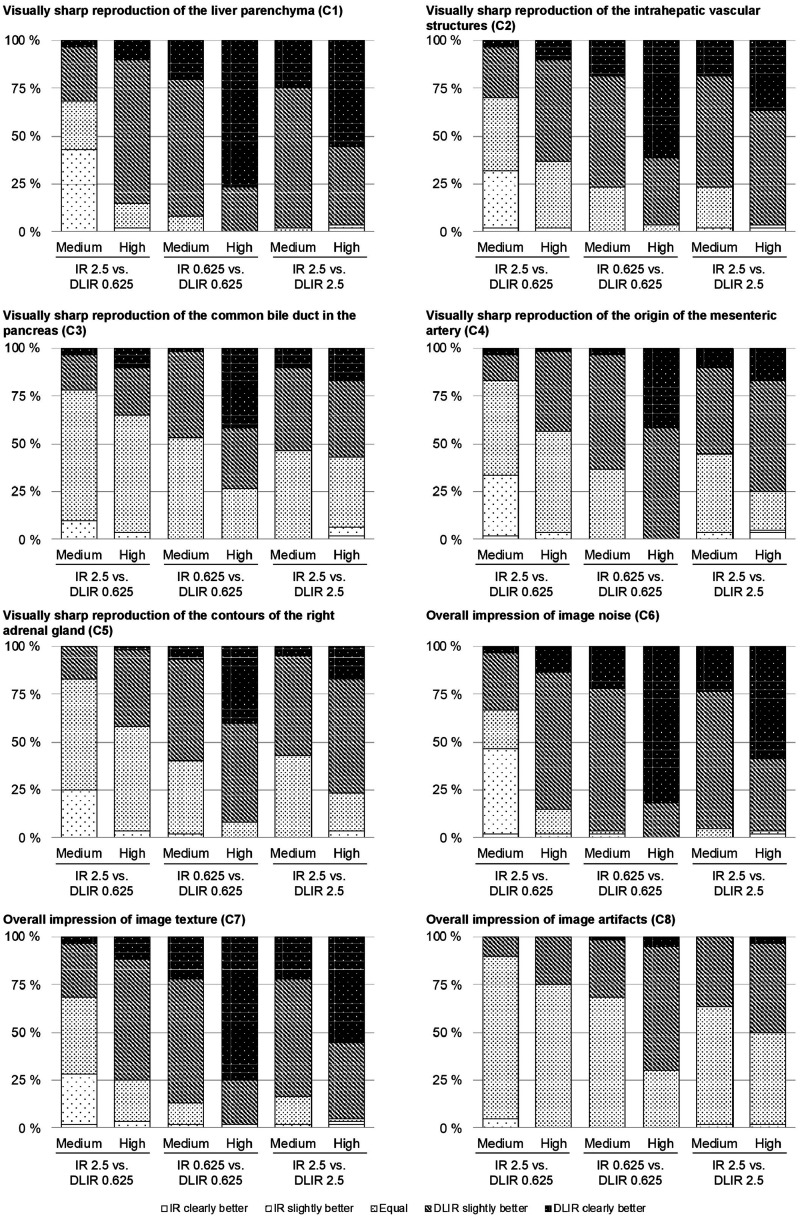
Distribution of visual grading scores assigned by three radiologists along eight visual grading criteria. Images reconstructed with DLIR were more frequently perceived as slightly better or clearly better compared to IR for all six image comparison sets, except the set comparing 0.625 mm DLIR of medium strength with 2.5 mm IR.

**Fig. 2.**
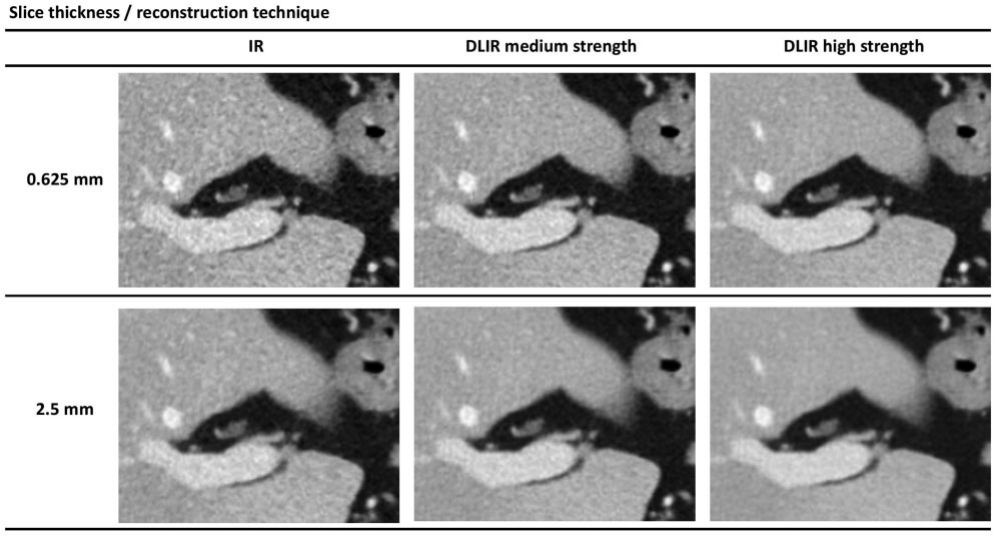
A selection of axial CT of the abdomen images centered on the liver hilum, reconstructed with standardly applied IR, DLIR of medium strength and DLIR of high strength in 0.625 mm and 2.5 mm slice thickness.

For visual grading analyses, DLIR image quality was more frequently perceived as *slightly better* or *clearly better* across all visual grading criteria compared to IR for all six image comparison sets, except the set comparing 0.625 mm DLIR of medium strength with 2.5 mm IR (Fig. 1). When dichotomizing visual scores as DLIR perceived as *equal* or *better* than IR (score of 0, 1 or 2 in favor of the images reconstructed with DLIR) as opposed to worse (score of 1 or 2 in favor of images reconstructed with IR), DLIR was significantly more frequently perceived as equal or better than IR for all visual grading criteria across all tested image sets, except for a selection of criteria in the set comparing 0.625 mm DLIR of medium strength to 2.5 mm IR (Table 2). Noteworthy, when evaluating DLIR perceived as *better* as opposed to *equal* or *better* for IR, DLIR was significantly more frequently perceived as better for visual reproduction of liver parenchyma (C1), visually sharp reproduction of the intrahepatic vascular structures (C2), overall impression of image noise (C6) and overall impression of image texture (C7) for all compared sets, except for 0.625 mm DLIR of medium strength to 2.5 mm IR (Table 3). A selection of axial CT images across reconstruction techniques are presented in Fig. 2.

For assessment of interobserver and intraobserver agreement, the categories *slightly better* and *clearly better* were merged to further emphasis contradicting scores between readers on which reconstruction technique was perceived better (for interobserver agreement), or contradictions by the same reader when viewing images of the same patient (for intraobserver agreement). For interobserver agreement, the two-way fixed reader average ICC(2.3) was estimated to 0.58, with 95% CI 0.53–0.62 (p < 0.001), collectively indicating moderate interobserver reliability.^15^ Noticeably, ICC for the five-point nominal scale was slightly higher, estimated to 0.68 (95% CI 0.64–0.71, p < 0.001). When evaluating intraobserver agreement on the three-point scale, the collective proportion of observed agreement was 75.7% (1090 of the 1440 pairwise visual scores). Bias index was 3.6% and Cohen’s Kappa was estimated to κ = 0.50 (unweighted) and 0.54 (weighted) (p < 0.001), indicating moderate agreement.^16^

Results from quantitative analyses are presented in Table 5. Noise was significantly lower in the portal vein and liver parenchyma for images reconstructed with DLIR compared to IR, for both 0.625 mm and 2.5 mm slices (all p < 0.001). Measured in the liver parenchyma, mean noise reduction on 2.5 mm slices was 16% when applying DLIR of medium strength and 39% when applying DLIR of high strength. Comparing 0.625 mm slices with DLIR of high strength with 2.5 mm IR, a mean noise reduction of 12% was observed (p < 0.001 for difference). Furthermore, CNR between the portal vein and the liver parenchyma was significantly higher in images reconstructed with DLIR of medium and high strength (all p < 0.01), where mean CNR measurements for images with 2.5 mm slice thickness were 41% higher for DLIR of medium strength and 260% higher for DLIR of high strength, compared to IR.

**Table 5.** Mean CT-number, noise, and contrast-to-noise ratio (CNR) measurements in the portal vein (PV) and liver parenchyma for images reconstructed with Iterative Reconstruction (IR) and Deep Learning Image Reconstruction (DLIR) of medium and high strength.

Reconstruction technique	CT-number (HU)				Noise (HU)				Contrast-to-noise ratio	
	Liver	p-value	PV	p-value	Liver	p-value	PV	p-value	Liver to PV	p-value
0.625 mm slice thickness									
IR	121.5	–	173.1	–	16.5	–	18.4	–	13.7	–
DLIR Medium	121.7	0.600	174.2	0.011	13.0	<0.001	15.2	<0.001	23.1	0.006
DLIR High	121.8	0.536	174.3	0.010	9.7	<0.001	11.5	<0.001	41.3	0.005
2.5 mm slice thickness									
IR	121.5	–	173.1	–	11.0	–	12.5	–	24.3	–
DLIR Medium	121.5	0.877	174.1	0.009	9.3	<0.001	11.1	<0.001	34.3	0.008
DLIR High	121.7	0.682	173.9	0.050	6.7	<0.001	8.6	<0.001	63.5	0.006

Values are reported as mean, except for noise where a pooled standard deviation is reported. P-values for comparison with IR, applying a pairwise Student’s *t*-test. DLIR: Deep Learning Image Reconstruction; IR: Iterative Reconstruction.

## Discussion

This study demonstrates that image quality in abdominal CT is markedly improved when applying a novel DLIR technique compared to standardly applied IR technique. This is shown for a selection of quantitative metrics as well as perceived image quality among three radiologists according to tailored visual grading criteria. Noticeably, qualitative image quality was explored both according to anatomical criteria based on established guidelines for image quality in abdominal CT, as well as subjective assessment of image noise, image artifacts and image texture. Interestingly, reader scores generally favored images reconstructed with high-strength DLIR, providing images with lowest degree of noise. Furthermore, DLIR showed robust noise reduction with significantly improved CNR measurements between the portal vein to adjacent liver parenchyma.

Sufficient image quality to detect perhaps subtle anatomical changes suggestive of disease is paramount in medical imaging. Traditionally, producing higher quality CT images generally incurs an increase in radiation dose delivered to the patient where benefit is offset by radiation-related risks. Thus, an important part of CT technology development is to improve image quality while maintaining stable radiation exposure, or preserving sufficient image quality despite dose-reduction, exemplified by the clinical introduction of IR.^17^ As noted introductory, recent reports have shed light on important limitations of IR, specifically related to low-contrast detectability tasks such as detection of liver metastases or pancreatic masses when radiation dose is reduced below a certain threshold.^8^ Thus, CT images may appear diagnostically acceptable but fail to show important clinical information.

Coined a new era of image reconstruction, vendor-specific reconstruction engines based on deep learning are now emerging (TrueFidelity, GE Healthcare; AiCE, Canon Medical Systems). The DLIR technique strives to improve image quality in previously challenging areas, such as low-dose imaging, high-resolution imaging, and the evaluation of obese individuals. Phantom studies have shown that the DLIR technique can markedly reduce image noise, while maintaining noise texture and spatial resolution.^18–20^ However, although promising, the technology has yet to be thoroughly evaluated in clinical studies to assess image quality and ensure that diagnostic performance is not impaired when pursuing dose reduction.^8^

Interestingly, when comparing high-strength DLIR in 0.625 mm slices with IR in 2.5 mm slices, DLIR was more frequently perceived to have equal or better image quality for all study visual grading criteria. This has several important implications. First, this suggests that reconstructing in thinner slices with DLIR has the potential to enhance spatial resolution without compromising perceived image quality, although this may potentially be offset by a deterioration of image contrast. Second, reconstruction with isotropic 0.625 mm voxels has the benefit of more readily being able to visualize structures by use of 3D models or out-of-plane reformatting. Finally, the findings of preserved (or improved) image quality when reconstructing images in 0.625 mm slices with DLIR of high strength compared to 2.5 mm with IR can also suggest that significant dose-reduction may be feasible for 2.5 mm slices with DLIR of high strength while maintaining acceptable image quality, although not explicitly demonstrated in this study. If confirmed in future studies, and without compromising diagnostic quality, this may be especially attractive in a screening setting, pediatric imaging, and for patients in the need of repeated CT examinations.^17^

A few other studies have evaluated image quality in CT reconstructed with the DLIR technique in a clinical setting. A study by Jensen et al.^10^ investigated image quality in abdominal CT where images were reconstructed with TrueFidelity (GE Healthcare) of various strength and compared to IR (ASIR-V 30%). Demonstrated by reader scores for two radiologists, scores for *overall image quality* and *overall lesion diagnostic confidence* were progressively higher for higher strength of DLIR, and significantly higher than scores for IR. Noticeably, scans were conducted in a setting of high-dose oncological imaging. Another study by Park et al.^21^ compared lower extremity CT angiography reconstructed with ASiR-V (80% and 100%) with DLIR of low, medium and high strength, demonstrating significantly improved subjective image sharpness for the DLIR images. The authors conclude that high-strength DLIR was the most balanced image in terms of image noise and sharpness in an arterial contrast phase setting. The current study is thus an important supplement, demonstrating improved image quality along tailored anatomical visual grading criteria in addition to assessing image noise and texture, for both thick and thin slice images acquired in the portovenous contrast phase. A recent study by Akagi et al.^22^ investigated ultra-high-resolution CT (U-HRCT) images reconstructed with a Deep Learning Reconstruction (DLR) technique (AiCE, Canon Medical Systems), assessing *vessel conspicuity* and *overall image quality*, and comparing DLR images to both IR and MBIR. Scores for *overall image quality* were significantly higher for the DLR technique, whereas scores for *vessel conspicuity* were highest for MBIR. Interestingly, the study was conducted on CT images with 0.25 mm slice thickness, although not compared to images with higher slice thickness. In this regard, the findings of improved image quality of 0.625 mm slices reconstructed with DLIR of high strength compared to IR in 2.5 mm slices are especially interesting, despite being demonstrated from a different vendor-specific technique.

This study is not without limitations. First, although significant results, this study reflects an initial experience with the DLIR technique applied to a small number of patients. However, the findings strongly support improved image quality in the CT images reconstructed with the DLIR engine, and are further strengthened by arguably acceptable levels of intraobserver and interobserver agreement among readers. Larger clinical studies, preferably exploring image quality across dose levels, should be conducted to further explore the potential benefit of the DLIR technique. This is an area of active research within our group being pursued as a follow-up to this study. Second, although CNR measurements between vascular structures and liver parenchyma are validated in literature,^23,24^ it is yet to be determined whether this observed increase in CNR achieved with DLIR improves lesion detection in practice. This should be augmented by low-contrast detectability studies to further determine the DLIR diagnostic performance. Third, although promising results from visual grading of anatomical structures, prospective studies evaluating pathology in a diagnostic setting across clinical indications are needed to further explore the clinical benefit. Finally, this study did not compare the DLIR images to images reconstructed with traditional FBP. However, one would not expect these to have better performance characteristics than standardly applied IR.

In conclusion, this study shows that a novel DLIR technique improved image quality characteristics when compared to traditional IR for both quantitative and qualitative image quality metrics. Interestingly, DLIR of high strength reconstructed in thin 0.625 mm slices was perceived to have better image quality when compared to conventional IR with 2.5 mm slices. As these findings represent an initial clinical experience, additional studies are required to further explore image quality properties of the DLIR technique to evaluate diagnostic performance across dose levels.
